# Pre-exposure Prophylaxis: The Delivery Challenge

**DOI:** 10.3389/fpubh.2018.00188

**Published:** 2018-07-06

**Authors:** Willem D. F. Venter

**Affiliations:** Wits Reproductive Health and HIV Institute, Faculty of Health Sciences, University of Witwatersrand, Johannesburg, South Africa

**Keywords:** pre-exposure prophylaxis, PrEP, HIV, prevention, operational, service delivery

## Abstract

HIV pre-exposure prophylaxis (PrEP), despite its efficacy, has been slow in uptake throughout the world. This article discusses the operational and service delivery complexity, based on early real-world experience, with some recommendations on how this promising intervention could be made more available. Currently, access to PrEP is made complex by restricting delivery to dedicated health facilities, as well as requirements around HIV testing and toxicity monitoring. Expanding to verticalized services that focus on well-defined populations where HIV testing and treatment programmes already exist, has proven relatively simple. Providing PrEP outside of this environment has yet to be demonstrated at any scale, and will likely require innovative thinking addressing the regulatory and clinical environment, so as to ensure easier access to this promising HIV prevention tool.

## Introduction

Oral pre-exposure HIV prophylaxis (PrEP) is highly effective in preventing HIV in adherent, high-risk individuals, and is well-tolerated (1–3). However, the intervention is complex for health systems to deliver, frustrating impatient policy makers, activists and researchers, faced with few effective HIV prevention techniques (3–6). These reasons are complex, and have evolved in the last few years; initially the cost of the intervention was a major barrier in richer countries, and while this remains an issue, programmes have meant greater access to the medication. However, cost has proved only one of many challenges that include issues as broad as providing laboratory monitoring that forces regular clinic attendance to discomfort with the health system in dealing with issues relating to sex. Understanding these challenges allows service delivery programmes for this very promising technology to be designed to be as cost-efficient and effective as possible.

Despite being shown to be effective since 2010, PrEP has only been rolled out at some scale in the USA, largely among men-who-have-sex-with men (MSM) (7). In focused epidemics, such as the UK, PrEP appears to have been partially responsible for a substantial drop in new infections (8). In many countries in Europe, Australia, Asia and Northern America, where PrEP has been prohibitively expensive, self-importation has emerged, as well as informal “buyers clubs” have arisen, that procure generic PrEP internationally, and return the drugs to groups requesting PrEP in-country (9). However, the burden of ongoing new infections reside in Africa, with a disproportionate number among heterosexual young women (10).

This article will examine some of the systemic delivery issues that face countries implementing PrEP, especially poorer countries with weak health systems, drawing from implementation experience with programmes addressing high risk populations. This includes the construction of guidelines and technical support to governments and policy makers, while providing training, and administering PrEP to sex workers, truck drivers, community members and adolescents in Southern Africa in the last 3 years. The article restricts discussion largely to oral PrEP and does not examine possible emerging PrEP technologies that include injectable and vaginal ring prophylaxis, as these are as yet not available, being in various phases of clinical trials (3). However, much of what is discussed below regarding oral PrEP is likely to apply to these technologies, if and when they become broadly available.

## Perceptions of adherence

Adherence to PrEP is frequently conflated with adherence to antiretroviral therapy (ART) for HIV infection, a bar that has traditionally been very high, by health care workers and programme managers (11). ART use with current WHO-recommended regimens requires over 90% adherence to prevent the development of resistance, and health care workers have had this messaging instilled since the inception of widespread HIV provision 15 years ago. In addition, messaging is that ART should never be interrupted, once initiated. This conflation is presumably because both ART and PrEP involve antiretrovirals (often the same antiretrovirals) (12).

Messaging on adherence for PrEP is far more nuanced, as it almost always involves interruptions. People move in and out of “cycles of risk,” where continuing PrEP is a waste of time with unnecessary exposure to the medication. A sex worker may interrupt her PrEP when she returns home to her rural area for a long holiday. A gay man may decide on a period of abstinence, after entering college and intensive study. A young woman may get agreement on a monogamous relationship with a new partner. People taking PrEP recognize this decreased risk, within programmes, and stop their medication. Taking PrEP when there is no risk-taking behavior is medically unnecessary and irrational, yet programmes are often measured against “loss to follow up,” similar to treatment programmes, and the movement off PrEP when leaving a cycle of risk is reported as a programme failure. In addition, health workers have a long tradition of admonishing patients who are “non-compliant” or interrupt therapy in other chronic diseases, and this has been seen in some of our programmes with PrEP.

Solutions to these issues vary, and include better success metrics for service provision of PrEP, to allow managers to have a better understanding of success and failure of PrEP programmes. For front-line health workers, better training is needed that emphasizes PrEP is more akin to contraception than ART treatment, and that moving off and on PrEP is completely acceptable, and even desirable, for patient care.

## Uptake of PrEP

Even within the US, PrEP use increased slowly initially. Part of this was cost, but even within subsidized programmes, initial uptake was slow, partly due to lack of marketing within the MSM community, a lack of access sites, concerns about side effects, and perceived awkwardness in conversations with health workers (7, 13). A similar slowness was seen within a sex worker programme in Johannesburg; interestingly, this accelerated once PrEP was registered by the regulatory authority in South Africa (14). Sex workers reported that they felt less like research subjects, and that the registration had legitimized the intervention as conventional medical practice. These perceptions among patients are important, and social marketing of the intervention appears to be imperative.

Slow access to novel interventions is not new in the HIV prevention world, and simply making an intervention available is unlikely to result in widespread uptake. Condomsand male circumcision programmes, within HIV prevention programmes that made these available, required extensive and ongoing marketing to encourage and facilitate access. Both of these interventions are different from but arguably simpler interventions than PrEP. Clear messaging regarding who should take PrEP, and how it can be accessed, along with relying on patient and social media word-of-mouth is necessary, along with reliable information on efficacy and side effects. Within the sex worker PrEP programme in Johannesburg, conflation with ART programme treatments meant side effects associated with ART efavirenz-containing treatment (dizziness, vivid dreams) became cited as side effects for sex workers on PrEP. Among health care workers, concerns about issues such as antiretroviral resistance and encouraging sexual disinhibition can often subtly translate into discouragement of the intervention.

## Getting prep to those who need it

PrEP programmes have addressed key groups such as sex workers, intravenous drug users and MSM; however, the critical group that requires intervention within Southern Africa is young women, and PrEP programmes addressing them have only recently been developed (15). These programmes, while ambitious, have proved to be very complex to deliver, again for various reasons. These include provision of care outside of the school environment, due to perceived resistance from school management and parents. Identifying young women easily and efficiently has proved surprisingly challenging, even within verticalized programmes addressing young sex workers.

Finding effective mechanism to deliver PrEP to young women, both in and out of school, will be key for HIV epidemic control. Understanding adherence problems, a concern seen with some PrEP research programmes involving women, is another challenge facing programmes (3).

## Integration within normal clinic flow

Vertical programmes that routinely offer HIV testing to high risk groups, such as those to sex workers, MSM and intravenous drug users, generally find the implementation of PrEP relatively easy. However, in routinely overburdened primary care servicing the general population with a wide variety of clinical conditions, the time-consuming nature of the discussion and administration of PrEP to healthy, asymptomatic people means it does not integrate well within routine services. In addition, several issues found within clinical service flow make PrEP provision challenging.

### Talking about sex

Health care workers are drawn from very religious societies in African countries, and moral concerns about sex are often conflated with clinical advice, in issues such as contraception, HIV testing, other sexually transmitted diseases, and now PrEP (16) (the author was part of a Swaziland consultation where religious leaders, alongside Ministry of Health officials, referred to PrEP as the “promiscuity pill”). Health care workers internationally, not just in Africa, have difficulty with the intimacy of sexual behavior discussions, and rarely have formal training in introducing these discussions. Slowing down clinical flow to allow for these conversations would require additional health care workers.

Disinhibition concerns figure large in health care workers' concerns about administration of PrEP—especially that it will lead to reckless sexual behavior and decreased condom use, despite no evidence from clinical research that this is the case. However, these concerns may lead to overt and more subtle discouragement of uptake of PrEP—again, a pattern seen with contraception programmes in the past.

### Monitoring

The routine blood monitoring that is required from current PrEP programmes is a major operational complexity (12). At the least, regular HIV testing is required. This is to ensure that non-suppressive ART (as used in the two drug PrEP combination) is not maintained if a person develops “breakthrough” HIV on the PrEP regimen, as this will lead to antiretroviral resistance. Breakthrough infections should be treated with three-drug regimens as quickly as possible, to prevent immune damage. This regular HIV testing is usually provided by the initiating programme, and in most cases requires a visit to a health facility and provider. Testing is a major cost driver of programmes, and places substantial burden on health care workers.

HIV self-testing is an exciting but expensive adjunct (often 2–3 times more expensive than the HIV tests used in clinics) to PrEP programmes, potentially meaning that people taking PrEP could self-screen at home (17, 18). In richer countries self-tests can cost as much as $40; in lower income countries, price guarantees and promises of large volumes have meant the price is likely to be below $10 a test (and possibly only $1–3 for large government contracts), but this is substantially more than < $1 for current “rapid” tests used within clinics. However, concerns have recently been aired that these technologies may lose sensitivity to detect infection in “breakthrough” infections (18, 19).

Current PrEP utilizes tenofovir, which is rarely a cause of renal toxicity, a serious side effect, especially in places where diagnostic and treatment facilities for renal disease are limited. A screening renal test (the creatinine clearance) is cheap and easily integrated within the HIV testing algorithm. No cases of renal failure have yet been seen after a normal screening test, using tenofovir-containing PrEP, but most guidelines recommend continuing monitoring (12, 17). The field needs to consider whether this is truly necessary, as it introduces yet another blood draw and visit to the clinic, as well as interfering with self-testing initiatives. The same holds for hepatitis B, where withdrawal of tenofovir in those with chronic replicative hepatitis B, has been associated with isolated anecdotes of “flares” (12).

### Hidden time and financial burden of clinic attendance

Regular clinic attendance, and the travel and time away from work that often accompanies PrEP programmes, may become an access issue, as they are for other chronic diseases and ART programmes. The vertical provision of contraception was stopped within South Africa, in an effort to “integrate” primary health care services, and led to decreased use of contraception as women suddenly had to wait for far longer for access to health care workers, rather than being able to dependably and speedily get their injectable or oral contraceptive. Efforts to make PrEP more widely available will have to be balanced with making it more easily accessible, as these are not synonymous.

## Alternative PrEP delivery sites

Alternatives to clinics are being explored, including using community based organizations and pharmacies. Provision at this level may be limited by legal restrictions regarding the prescription of PrEP, but deserve exploration to facilitate access. The drugs used are very safe, but the requirements of blood testing may limit where the intervention can be accessed. Innovative methods of both collecting laboratory samples, as well as drug delivery, may obviate unnecessary visits to clinical services.

## Conclusions

PrEP is new, and health care workers require training and experience to become comfortable with the intervention. Efforts are needed to simplify provision of the intervention, to make delivery as efficient as possible. Health metrics need to be more sophisticated in assessing levels of success with PrEP programmes. Vertical programmes, where implementing PrEP is relatively simple, are ideal sites for rapid expansion of PrEP to priority groups, but integration within routine primary care requires substantial creative thinking and programme design.

## Author contributions

The author confirms being the sole contributor of this work and approved it for publication.

### Conflict of interest statement

The author declares received drug donations for PrEP and treatment trials from Gilead Sciences, who developed currently used oral PrEP.
